# Translating a Global Emission-Reduction Framework for Subnational Climate Action: A Case Study from the State of Georgia

**DOI:** 10.1007/s00267-020-01406-1

**Published:** 2021-01-15

**Authors:** Marilyn A. Brown, Blair Beasley, Fikret Atalay, Kim M. Cobb, Puneet Dwiveldi, Jeffrey Hubbs, David M. Iwaniek, Sudhagar Mani, Daniel Matisoff, Jaqueline E. Mohan, Jeffrey Mullen, Michael Oxman, Daniel Rochberg, Michael Rodgers, Marshall Shepherd, Richard Simmons, Laura Taylor, L. Beril Toktay

**Affiliations:** 1 School of Public Policy, Georgia Institute of Technology, Atlanta, GA 30332 USA; 2 Rollins School of Public Health, Emory University, Atlanta, GA 30322 USA; 3 School of Earth and Atmospheric Sciences, Georgia Institute of Technology, Atlanta, GA 30332 USA; 4 Warnell School of Forestry and Natural Resources, University of Georgia, Athens, GA 30602 USA; 5 Urban Studies Institute, Georgia State University, Atlanta, GA 30303 USA; 6 School of Chemical, Materials & Biomedical Engineering, University of Georgia, Athens, GA 30605 USA; 7 Odum School of Ecology, University of Georgia, Athens, GA 30607 USA; 8 College of Agricultural and Environmental Sciences, University of Georgia, Athens, GA 30602 USA; 9 Scheller College of Business, Georgia Institute of Technology, Atlanta, GA 30308 USA; 10 School of Civil and Environmental Engineering, Georgia Institute of Technology, Atlanta, GA 30332 USA; 11 Department of Geography, University of Georgia, Athens, GA 30602 USA; 12 Strategic Energy Institute, Georgia Institute of Technology, Atlanta, GA 30332 USA; 13 School of Economics, Georgia Institute of Technology, Atlanta, GA 30332 USA

**Keywords:** Carbon footprint, Carbon neutrality, Equity, Climate roadmap

## Abstract

Subnational entities are recognizing the need to systematically examine options for reducing their carbon footprints. However, few robust and comprehensive analyses are available that lay out how US states and regions can most effectively contribute. This paper describes an approach developed for Georgia—a state in the southeastern United States called “Drawdown Georgia”, our research involves (1) understanding Georgia’s baseline carbon footprint and trends, (2) identifying the universe of Georgia-specific carbon-reduction solutions that could be impactful by 2030, (3) estimating the greenhouse gas reduction potential of these high-impact 2030 solutions for Georgia, and (4) estimating associated costs and benefits while also considering how the solutions might impact societal priorities, such as economic development opportunities, public health, environmental benefits, and equity. We began by examining the global solutions identified by Project Drawdown. The resulting 20 high-impact 2030 solutions provide a strategy for reducing Georgia’s carbon footprint in the next decade using market-ready technologies and practices and including negative emission solutions. This paper describes our systematic and replicable process and ends with a discussion of its strengths, weaknesses, and planned future research.

## Introduction

To avoid the worst impacts of a changing climate, more than 190 countries agreed to the Paris Agreement goal of limiting global temperature rise to below 2 °C (3.6 °F) above the preindustrial global average and to attempt to achieve a 1.5 °C (2.7 °F) target. The Intergovernmental Panel on Climate Change (IPCC) estimates that achieving these targets would require net-zero global emissions of greenhouse gases (GHG) by 2070 and 2050, respectively, and rapid action by national and subnational economies across the globe (IPCC 2018).

Most detailed analyses of pathways for achieving economy-wide emission reductions are at the global or national scale. US regions and states cannot easily convert these larger-scale studies into playbooks for local action, and political dynamics in some states have prevented comprehensive carbon planning. Carbon planning will have to be flexible in its implementation to address local contexts and issues. While global and national studies provide a powerful point of departure, they must be tailored to meet the needs, resources, economies, and capabilities of specific localities and their potentially different priorities and preferences.

This paper describes one novel effort to translate the global framework developed by Project Drawdown (Hawken 2017; Frischmann et al. 2020) to a local set of solutions for reducing net emissions over the next decade in Georgia—a state in the southeastern United States that has not yet developed a state-wide emission-reduction plan. This approach seeks to identify high-impact, cost-competitive solutions to reduce carbon emissions—and identify the associated economic, environmental, equity, and health impacts and benefits. In this paper, we describe (1) our replicable methodology that uses a subnational lens to examine the global Project Drawdown solutions, (2) the results of the analysis as applied to Georgia, (3) the strengths and weaknesses of our methodology, and (4) planned next steps.

As described below, the framework starts with a review of climate impacts in the state and then an examination of the baseline of the state’s consumption of fossil fuels, the energy requirements of its end-use sectors, and its GHG emissions. Surveys and other public outreach was conducted at multiple points in the research process to elicit preferences of the state’s residents and experts. Finally, a systematic approach was developed and applied to identify which of the global emission-reduction solutions highlighted by Project Drawdown provide the most promising opportunities to reduce net GHG emissions over the next decade in Georgia. In addition, this paper describes how “beyond carbon” priorities—such as equity, public health, economic development, and the larger environment—were included as part of the methodology.

## Global and National Frameworks for Emission Reductions

A recent World Meteorological Organization (WMO) report concluded that there is roughly a 70% chance that 1 or more months during the next 5 years will exceed preindustrial levels by 1.5 °C or more (WMO 2020). These findings, issued in the WMO Global Annual to Decadal Climate Update, are based on climate predictions and recent trends in global temperature observations. Seneviratne et al. (2018) describe possible climate outcomes from the 1.5 °C target established in the Paris Agreement. Their work confirms that the basket of emission-limiting solutions for achieving the 1.5 °C warming goal can mitigate against risks associated with higher levels of global warming. However, they warn that none of the scenarios guarantee avoidance of larger climate risks at regional scales such as the Southeastern United States, given that some regions may experience warming trends greater than the global average.

The scale and complexity of climate change mitigation requires multilevel governance, capacity building, and cross-sector changes on local, national, and global scales (Daniell et al. 2011; Di Gregorio et al. 2019; Alves et al. 2020). Project Drawdown is one of many approaches that have been used to identify strategies for reducing GHG emissions on a global scale. As part of the Paris Agreement (2015), the IPCC was invited to analyze pathways for limiting global temperature rise to 1.5 °C. The IPCC analysis considered a range of modeling scenarios that highlight the need to cut emissions in all sectors of the economy, including land, energy, industry, buildings, transportation, and cities (IPCC 2018). Other groups, such as Princeton University, have also analyzed pathways for reducing global GHG emissions. Princeton’s Stabilization Wedge Framework (https://cmi.princeton.edu/wedges) considers how deploying a portfolio of existing technologies can collectively keep global emissions from rising while meeting projected growth in global energy demand (Socolow and Pacala 2006). The effort highlights 15 strategies that each has the potential to reduce global carbon emissions by at least 1 billion tons per year by 2060. This includes strategies such as doubling fuel efficiency of 2 billion cars from 30 to 60 miles per gallon or installing 100 times the current capacity of solar electricity. Other approaches have been used by the United Nations emission gap reports (UN Environment Programme 2019) and the McKinsey cost curves (McKinsey and Company 2020).

Project Drawdown highlighted global solutions that could be deployed to achieve “drawdown”, or the point at which the concentration of GHGs in the atmosphere starts to decline (Hawken 2017). Its solutions spanned the “traditional” sectors and engineering technologies, such as retrofitting buildings, increasing solar power, and deploying electric vehicles. Project Drawdown also extended its focus to include an array of social–ecological–technological opportunities such as educating women and girls, adopting plant-rich diets, and reducing food waste that has not been part of the traditional IPCC carbon mitigation measures. The project also included options for capturing emissions through natural and technological sinks, such as reforestation, preserving coastal wetlands, and direct air capture. This work attracted the attention of global audiences with the release of Project Drawdown’s New York Times best-selling book in 2017. However, the work had not been translated into an actionable plan for a targeted community until Drawdown Georgia.

At the national scale, 186 countries have submitted national action plans to the United Nations Framework Convention on Climate Change (UNFCCC) in the form of “Nationally Determined Contributions” under the Paris Agreement (2015) (https://www4.unfccc.int/sites/ndcstaging/Pages/Home.aspx). In addition, in response to the Paris Agreement’s call for countries to identify “mid-term long-term low greenhouse gas emissions development strategies”, 39 countries, accounting for 73.5% of global emissions, have submitted a long-term decarbonization strategy to the UNFCCC (https://www.climatewatchdata.org/lts-explore).

Several observers have pointed to the critical role that subnational climate action plays in achieving national and global climate mitigation goals. Ostrom (2010) highlights the emergence of a polycentric approach to climate, with actions being taken at the household, organizational, municipal, and state level. Brown and Sovacool (2011) describe nine case studies of emission-reduction programs that illustrate the benefit of mixing traditional scales and engaging multiple actors. Blok et al. (2012) estimated the contribution that subnational governments can make to reducing global emissions. Jänicke (2017) highlights the sometimes “pioneering” role that subnational regions play in a system of multilevel climate governance by “experimenting and providing best practices”. The UNFCCC formally embraced subnational action as part of the global climate framework when it launched a Global Climate Action database in 2014; as of September 2020, this database included actions by 244 cities and 19 states in the United States (https://climateaction.unfccc.int/#US).

Within the United States, several states and cities have undertaken climate-planning efforts. Twenty-five member states of the US Climate Alliance (2019) have each adopted a range of state-level climate policies, and several states have developed detailed analyses of their carbon footprint and mitigation opportunities (http://www.climatestrategies.us/us-projects-programs). That said, these efforts have not sought to connect a global framework like Drawdown to the state-level context. This is a key contribution of this paper. In addition, we expand the Drawdown methodology by including local-level “beyond carbon” priorities—equity, public health, economic development, and broader environmental impact.

## Overview of Drawdown Georgia

The Drawdown Georgia project was initiated in 2019 to create a replicable framework for translating a global emission-reduction analysis to a subnational level. Our effort is a replicable, systematic approach to identifying key action levers at the state level. While this effort does not go so far as to translate these solutions to specific policies, it does rely on input from a broad range of stakeholders to provide insights on how to implement solutions that are sensitive and flexible to local priorities and capabilities—even in jurisdictions that have been resistant to economy-wide carbon policies.

For Drawdown Georgia, we assess the global solutions identified by Project Drawdown and highlighted the most promising opportunities to reduce net GHG emissions in the state of Georgia over the next decade. Net emissions refer to the difference between the release of GHGs from fossil fuels and other “sources”, as well as the sequestration of GHGs by ecosystems such as forests and coastal wetland plants and soils, and other “sinks”. This effort focused on identifying solutions best suited to reducing state-level emissions by 2030. However, the methodology could be applied to review solutions relevant to a longer time horizon.

Our framework starts by describing baseline climate impacts and GHG emissions and sinks in Georgia. As context for considering solutions over the next decade, we also review baseline forecasts of Georgia’s GHG emissions through 2030. A series of surveys and focus groups were conducted to elicit the preferences and insights of the state’s residents and experts. Then, a systematic approach was developed and applied to identify which of the 102 global emission-reduction solutions highlighted by Project Drawdown provide the most promising opportunities to reduce net GHG emissions over the next decade in Georgia. This approach is centered on a four-step downselection process that builds off of existing climate frameworks described above, to show how scaled-up deployment of individual climate solutions can collectively contribute to carbon-reduction goals:

- Step 1: Is the solution technology and market ready for the state?
- Step 2: Is there sufficient local experience and available data?
- Step 3: Does the solution provide meaningful emission reductions in the relevant timeframe?
- Step 4: Is the solution cost-competitive?

Finally, this paper describes how “beyond carbon” impacts and priorities—such as equity, public health, economic development, and the larger environment—were considered as part of the methodology. The primary purpose of identifying “beyond-carbon” factors early in the project was to ensure that they were identified throughout the downselection and evaluation process and incorporated into future pathway development and implementation stages.

We outline many of the specific steps below. However, we stop short of providing excessive detail on some aspects of the process, such as details on how to run public workshops or how to allot researchers’ time. This is to avoid being overly prescriptive, which could result in errant application or inappropriate prioritization of the process by another region or stakeholder effort.

## The Context for Climate Action in Georgia

The Southeast is susceptible to a broad spectrum of extreme weather and climate events, including drought, heatwaves, floods, hurricanes, tornadoes, and wildfires (Comou and Rahmsdorf 2012). Natural disasters in the southern states, in recent decades, have outpaced similar events across the United States annually in both magnitude and scale. Emrich and Cutter (2011) report ratios of almost 4:1 during the previous decade. In Georgia, such extremes have a direct impact on agricultural productivity, energy production, public health, infrastructure, transportation, and more (Rudd et al. 2018).

The Southeast is projected to experience more intense heatwaves and droughts in the future (Kunkel et al. 2010; IPCC 2014). Indeed, the National Climate Assessment (NCA 2018) has revealed that much of North America, including the Southeast, is now experiencing statistically significant increases in warm nights and a reduction in extreme cold. Specifically, average daily minimum temperatures are increasing at a rate three times faster than daily maximum temperatures (NCA 2018). The literature also finds more intense and frequent hydrometeorological extremes, expressed in terms of extreme rainfall rates as well as sustained deficits, consistent with climate model projections showing that extreme events on both tales of the rainfall distribution would increase in response to anthropogenic forcing (NCA 2018). Tropical cyclone intensity is likely shifting to atmospheric and oceanic warming as well (Knutson et al. 2015; Kossin et al. 2020), with a growing percentage of “major storms” (categories 4 and 5) over recent decades. In Georgia, Hurricane Michael caused over $2.5 billion in agricultural losses in 2018 (UGA Cooperative Extension), and Hurricane Irma caused $670 million in damages in 2017 (Senkbeil et al. 2020).

The low-lying Southeastern coastline is uniquely susceptible to ongoing sea-level rise, which poses an acute threat to the thriving, culturally rich communities of coastal Georgia. Sea levels have risen 10″ in the last 85 years, as measured at NOAA’s Fort Paluski tide gauge located in Savannah, Georgia, and are projected to increase by 1–4 feet by 2100 (68% probability range), although up to 10 feet of sea-level rise is possible under extreme scenarios (NCA 2018). When combined with increased frequency and intensity of tropical storms in the North Atlantic basin over recent years, sea-level rise has contributed to increased flooding along the Georgia coastline, with 69% of “major floods” occurring since 2015 (National Weather Service, Charleston).

Georgia is home to large populations of urban and rural poor, including historically marginalized African-American communities, who are uniquely vulnerable to a large range of climate-related stressors that exacerbate long-standing inequalities (Binita et al. 2015). Projected county-level economic losses across Georgia of up to 10% by 2100 reflect acute vulnerabilities from a combination of diminished agricultural yields, reduced access to high-risk labor, heat-related mortality, and coastal losses related to sea-level rise (Hsiang et al. 2017).

## Baseline Analysis of Georgia’s Energy Economy and GHG Emissions and Sinks

To generate projections of emission impacts for each technology, it was crucial to have an accurate accounting of Georgia’s baseline emissions as well as a business-as-usual projection of future emissions for the state. This section summarizes Georgia’s energy economy, its GHG emissions, sources of emissions, and its natural carbon sinks. The section ends by describing a forecast of Georgia’s projected GHG emissions in 2030, based on Georgia Tech’s National Energy Modeling System (GT-NEMS) and projections of the US Environmental Protection Agency’s (EPA) State Greenhouse Gas Inventory and Projection Tool (https://www.epa.gov/statelocalenergy/download-state-inventory-and-projection-tool).

We focus particular attention on Georgia’s energy economy because the combustion of fossil fuels is the largest source of the state’s carbon dioxide (CO_2_) emissions. All four sectors of Georgia’s economy are major consumers of energy and emitters of CO_2_—transportation, homes, businesses, and industry.

**Fig. 1:**
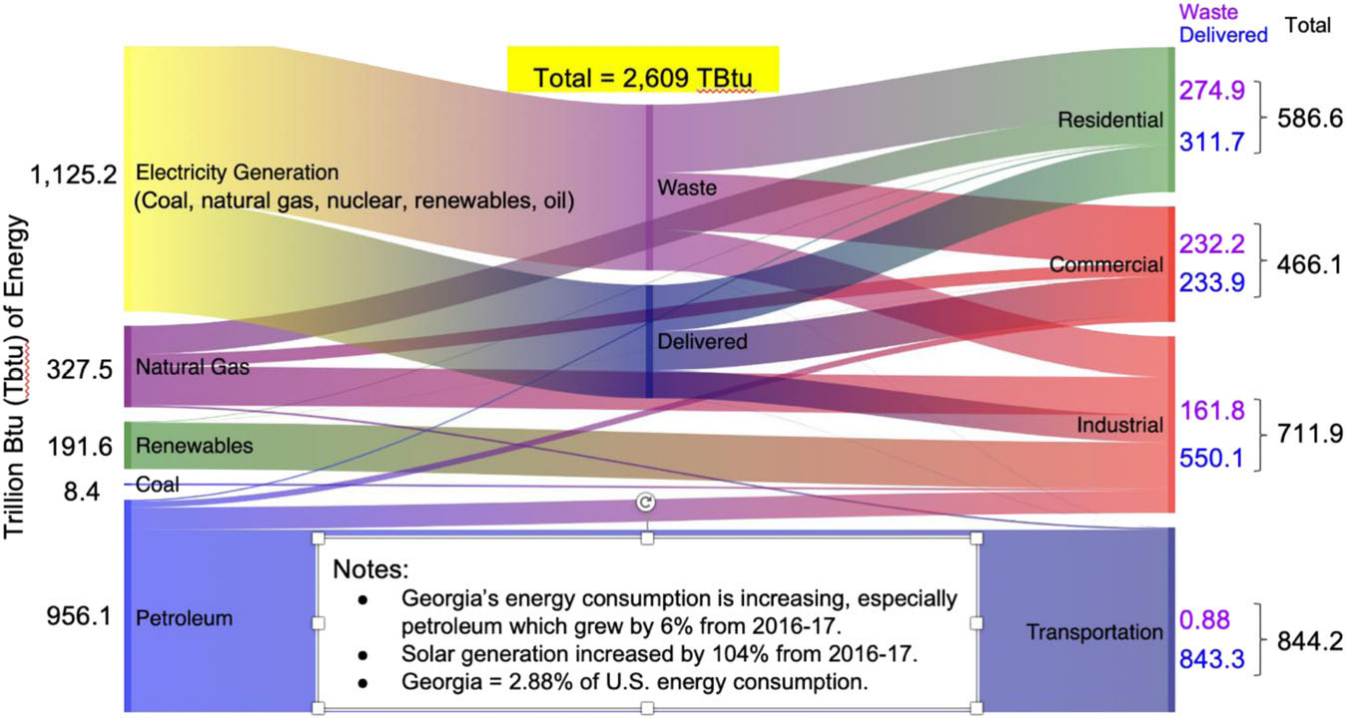
Georgia’s energy consumption in 2017. Source: authors, created with data from the Georgia Tech National Energy Modeling System (GT-NEMS) and https://www.eia.gov/state/seds/seds-data-complete.php?sid=GA#Consumption

In 2017, Georgia consumed 2609 TBtu of energy, accounting for 2.8% of US GDP and 2.9% of US energy consumption, indicating that the state’s economy is slightly more energy-intensive than the US economy. As Fig. 1 illustrates, the vast majority of this energy budget was spent on fossil fuels, dominated by petroleum (for transportation), natural gas (in electricity and industry), and coal (which was the dominant fuel for electricity generation in 2017, but it has recently been eclipsed by natural gas). Transportation is the largest consumer of energy in Georgia, followed by industry, homes, and businesses. This is the same rank order of energy use across sectors in the United States as a whole.

**Fig. 2:**
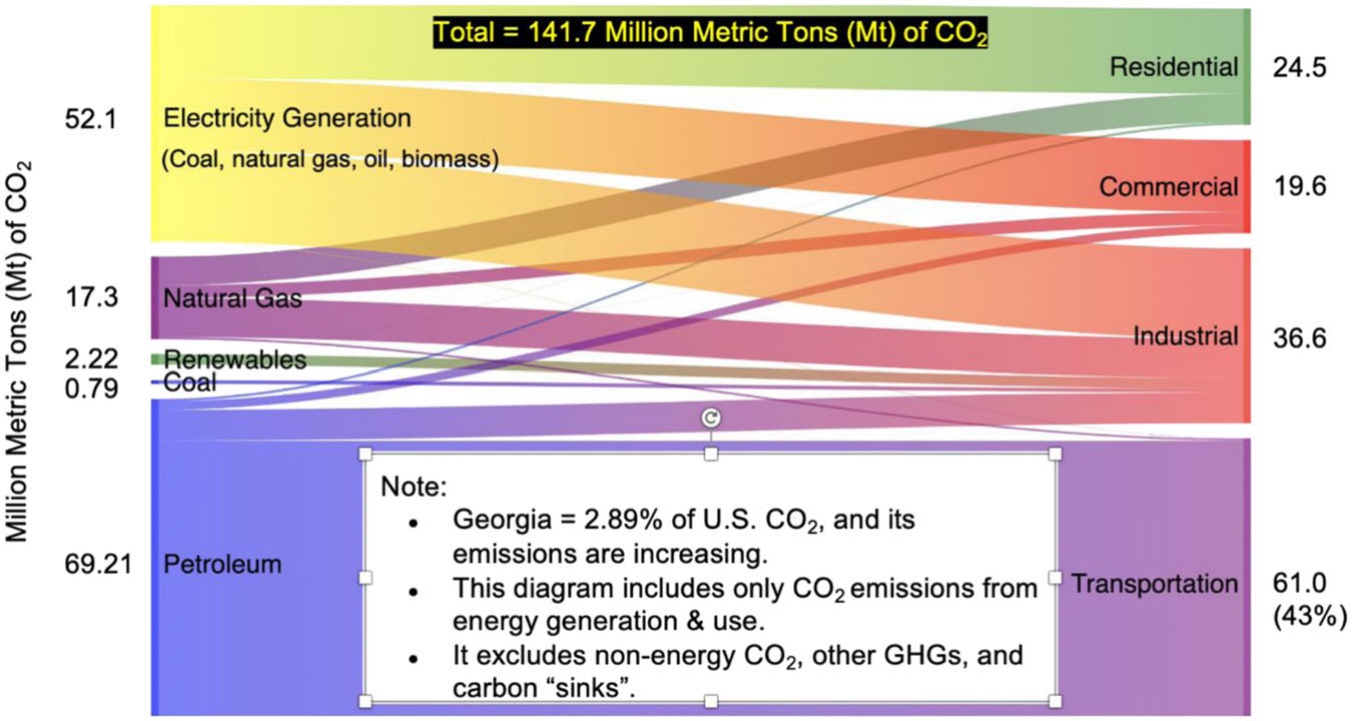
Georgia’s CO_2_ emissions from energy consumption in 2017. Source: authors, created with data from https://www.eia.gov/state/seds/seds-data-complete.php?sid=GA#Consumption; https://www.epa.gov/sites/production/files/2019-04/documents/2019_fast_facts_508_0.pdf. Values from the EPA “Fast Facts” website were used to estimate kg of carbon per million Btu, which was multiplied by 44/12 to estimate kg CO_2_ per million Btu for each of the fuels shown in Fig. 1

Georgia’s CO_2_ emissions from fossil fuel combustion totaled 141.7 Mt CO_2_ (or 141.7 “megatons”) in 2017 (Fig. 2), representing 2.9% of US emissions from fossil fuels.^1^ As with its energy intensity, this indicates that in 2017, the state’s economy was slightly more carbon intensive than the US economy. The dominant sources were transportation (at 69 Mt CO_2_) and electricity generation (at 52 Mt with 32 from coal and 20 from natural gas), suggesting that these sectors could be particularly productive targets for emission reductions.

Offsetting these emissions, Georgia has carbon sinks (or “negative emissions”), resulting from the uptake of CO_2_ in forests and agricultural soils. The World Resources Institute (WRI 2014) estimates an annual sequestration of roughly 46 Mt in Georgia in 2011. This is equivalent to about 32% of Georgia’s CO_2_ emissions from fossil fuels in 2017. Assuming that this value holds true in 2017, Georgia’s net carbon footprint would have been 108.8 Mt in 2017.

**Fig. 3:**
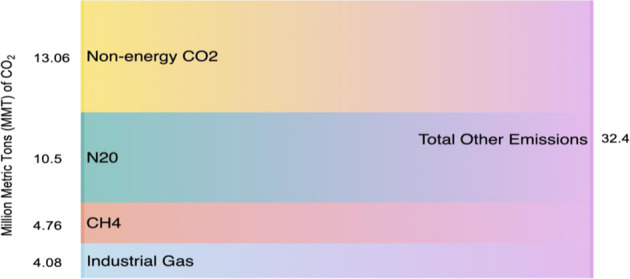
Georgia’s nonenergy CO_2_ and other GHG in 2017.^2^ Source: authors, created with data from https://epd.georgia.gov/air/sites/epd.georgia.gov.air/files/related_files/document/ghg_gainventory2012.pdf; https://www.epa.gov/sites/production/files/2019-04/documents/2019_fast_facts_508_0.pdf

In addition to CO_2_, there are several other sources of GHGs whose global warming potentials can be considered using standardized equivalency metrics called CO_2_-e (Fig. 3). EPA’s 2017 national GHG emissions inventory (EPA-2) (https://www.epa.gov/ghgemissions/overview-greenhouse-gases) estimated that Georgia emitted 174.1-Mt CO_2_-e, of which 6% was NO_*x*_, 2.7% was methane, and 2.3% was fluorinated gas. Altogether, the three non-CO_2_ sources of GHG emissions contributed to an estimated 19.3-Mt CO_2_-e or 11% of Georgia’s total GHG emissions. The remaining 89% of Georgia’s total emissions are from CO_2_.

In sum, Georgia’s net GHG emissions in 2017 are estimated to have been 128-Mt CO_2_-e: 142-Mt emissions from energy consumption plus 13 from nonenergy CO_2_ emissions plus 19 from three non-CO_2_ GHG emissions minus 46 Mt from carbon sinks.

To provide a baseline forecast of Georgia’s GHG emissions in 2030, we use GT-NEMS, a computable general equilibrium model of the US energy economy. GT-NEMS is the Georgia Institute of Technology’s version of the modeling system used by the US Energy Information Administration to produce its “2018 Annual Energy Outlook”. It therefore does not account for the Covid-19 pandemic, which has had far-reaching impacts on the US economy and society. In particular, the level of fossil fuel consumption and GHG emissions in 2020 and for some time in the future will be lower than these previous forecasts. Assuming a full recovery by 2030, GT-NEMS provides a reasonable point of comparison for considering the impact of alternative drawdown solutions in a decade’s time.

GT-NEMS forecasts GHG emissions from energy consumption for each of the 9 census regions of the United States. Georgia is located in the South Atlantic region, and it accounts for ~16.5% of the region’s economic activity measured along multiple dimensions, including population, state domestic product, retail electricity sales, and energy consumption. Using the GT-NEMS Reference Case forecast for 2030, we project that Georgia’s energy-based CO_2_ emissions will be 122 Mt in 2030. For comparison, the US EPA’s State Greenhouse Gas Inventory and Projection Tool (https://www.epa.gov/statelocalenergy/download-state-inventory-and-projection-tool) forecasts that Georgia’s energy-based CO_2_ emissions in 2030 will be 127.9-Mt CO_2_. GT-NEMS also offers insights about GHG emission trends and forecasts by sector. In 2030, CO_2_ emissions from energy consumption in Georgia are forecast to come up to 41% from electricity and 39% from transportation. Thus, clearly, these two sectors merit particular attention. Residential and commercial buildings are forecast to be responsible for 22% and 21% of energy-related CO_2_ emissions in 2030, much of which comes from their consumption of electricity. To round out the picture, industry (which includes the manufacturing of materials such as aluminum, chemicals, and paper) is expected to be responsible for 17% of energy-related CO_2_ emissions in 2030. These projections provide guideposts for considering the importance of different types of solutions.

## Survey of the Public and Experts

Engagement with the expert community and interested members of the public was weaved throughout the process through in-person and virtual collaboration. This helped to ensure that key issues are not overlooked.

The Drawdown Georgia team set the tone for this collaboration early in the research effort by hosting an Introduction to Drawdown Georgia Webinar. The webinar was held on August 2, 2019, and brought together 147 participants. In addition, we began to take public comments about the project on the Drawdown Georgia website (https://www.drawdownga.org) and invited residents to complete an online survey about possible Drawdown solutions. The survey was promoted through targeted emails, affiliate newsletters, and social media. A total of 280 respondents completed the survey, focusing on all, or a subset of the sectors, depending on their expertise and interests, ranging from 82 respondents for forest and land-use solutions to 98 respondents for electricity generation solutions. Their demographics show a wide range of participation by individuals living in Georgia, but also a bias toward affluence and education (Appendix).

**Fig. 4:**
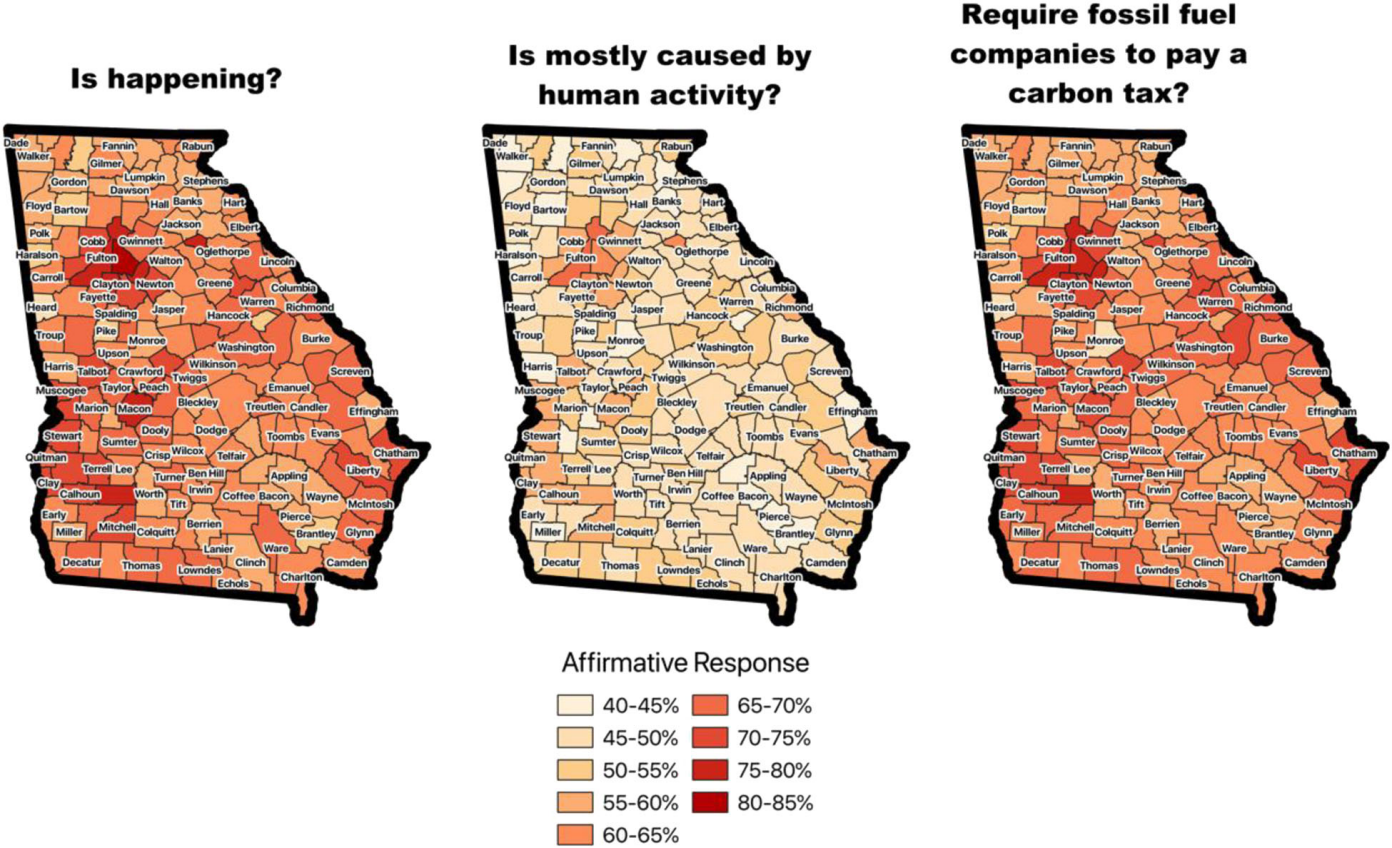
Public opinion regarding global warming in Georgia, 2019. Source: maps created by Drawdown Georgia from data provided by Yale and George Mason Universities (https://climatecommunication.yale.edu/visualizations-data/ycom-us/).

We also relied on existing public opinion research to survey preferences within the state. According to a 2019 Yale and George Mason University survey on climate change opinions (Leiserowitz et al. 2019), 72% of Americans think that global warming is happening and 59% believe that it is mostly human-caused. Drawdown Georgia’s county-level analysis of these data suggests that citizens across Georgia believe that global warming is happening, but the degree of certainty is lower in rural counties. Compared to the average American, Georgia residents are less certain that climate change is caused by human activity: in particular, a majority of residents in rural counties in Georgia do not agree that climate change is mostly caused by humans (https://climatecommunication.yale.edu/about/projects/global-warmings-six-americas/). Nevertheless, a majority of the survey respondents from Georgia are in favor of requiring fossil fuel companies to pay a carbon tax (Fig. 4).

## The Methodology for Downselecting Solutions for Georgia

The foundational step of defining the universe of solutions under consideration was conducted by six working groups comprising the faculty from the Georgia Institute of Technology, University of Georgia, Emory University, and Georgia State University. The working group leads and coleads created small teams of researchers, with the assistance of graduate students, who were assigned to examine the following six focus areas based on their areas of expertise: Electricity, Transportation, Built Environment and Materials, Food Systems, Land Sinks, and Beyond Carbon.^2^

The working group leads were selected and overseen by the Drawdown Georgia core team of seven researchers who include IPCC coauthors, a member of the National Academies of Engineering and Science, a retired State Department climate negotiator, and a Nobel Laureate. The six working group leads and coleads have hundreds of years of combined experience across the respective subject-matter domains. They conducted centralized (project-wide) and subject-matter- specific surveys covering > 200 experts, and conducted numerous meetings to engage stakeholders. The decision-making was filtered through a tiered structure that mirrored the project team organizational chart wherein working group leaders rely on rigorous justification and are accountable to the core team.

Expert opinions were also sought from subject specialists from other universities and in the government, nongovernmental organizations, as well as industry and business sectors. We held public and specialist conferences and surveyed both the public and specialists for detailed opinions about the merit of each potential solution. These opinions were compiled and used to inform the selection of the final solutions.

For example, the Land Sinks working group did not originally consider coastal wetlands as a viable final solution for our state, as the coastal lands in Georgia are limited in extent. However, given the very high CO_2_-e sequestration potential of these ecosystems, particularly in substrate pools that are better protected from disturbance than above-ground plant biomass pools, this solution was included in our final working group’s list of drawdown solutions. For this working group, the results from the public and the expert surveys were remarkably in line.

The Buildings and Materials working group used the expert focus groups and surveys to help discover additional technologies that were not originally considered. We used the experts and focus groups to narrow the retrofit category to find appropriate cost-effective technologies to model for various sectors.

Our process, by design, was both somewhat generalized (as per Project Drawdown), and somewhat customized (as per Drawdown Georgia). This, we believe, is one of our greatest contributions.

Drawdown Georgia developed a systematic and replicable methodology for downselecting the most promising solutions to meaningfully reduce net GHG emissions in the state over the next decade. This includes (1) defining the universe of solutions under consideration, (2) filtering solutions through a four-step downselection process, (3) identifying high-impact solutions, and (4) mapping beyond-carbon considerations. Each step is described below.

### Defining the Universe of Solutions under Consideration

Each working group began by examining all of the global solutions identified by Project Drawdown. The list was then reviewed in consultation with outside experts to determine (1) if additional solutions should be added to the mix or (2) if solutions identified by Project Drawdown should be defined differently to better fit the state of Georgia. For example, working groups identified solutions that, on their own, are unlikely to deliver meaningful emission reductions in Georgia over the next decade, but have the potential to contribute meaningfully if considered as a set. We call these “Bundled” solutions.

Bundled solutions include alternative mobility, recycling/waste management, retrofitting, afforestation and silvopasture, and temperate forest protection and management. Alternative mobility is one of the more complex bundles. When treated separately, telepresence, e-bikes, e-scooters, and walkable cities each only offer modest levels of carbon reduction by 2030. However, they could provide meaningful emission reductions by packaging them into an alternative mobility solution, which considers replacing emission-intensive vehicle miles traveled (VMTs) with one or more of these zero- or low-carbon alternatives. Walking and biking can replace short-distance vehicle trips, while teleworking can replace longer commuting trips.

Policies that impact carbon are unlikely to target one specific technology, but instead are likely to promote a suite of solutions or strategies in a particular sector. The bundles that we created are an attempt to group sets of technologies together that align with institutional approaches or policies to address carbon. Our retrofitting bundle, for example, incorporates a set of building improvements that might be addressed through a retrofitting program, and was expanded to include solutions beyond those included in Project Drawdown.^3^ Our alternative mobility bundle incorporates a set of infrastructure solutions and practices that might be addressed by a range of organizations and authorities managing commuting and urban design. These bundles were created as a direct result of our engagement with experts and the public.

In another instance, the solution highlighted by Project Drawdown is an enabler but not a direct contributor to carbon-emission reductions. Energy storage is one such solution. The analysis of solar farms, rooftop solar, and demand response includes the possibility of pairing solar panels with batteries to enable more impactful and cost-competitive solutions. Energy storage, therefore, was dropped as a stand-alone solution but is part of at least three bundled solutions.

After these additions and revisions, a total of 75 solutions were considered by Drawdown Georgia, which produced a short Georgia-specific assessment of each of these solutions (https://cepl.gatech.edu/sites/default/files/attachments/Drawdown_WPAppendix_041320.pdf).

### The Four-Step Downselection Process

Working groups put each of the 75 solutions through an initial qualitative and quantitative review. This included a literature review to define the solution further, identify Georgia-relevant data, establish the technology and market maturity of the solution, capture cost projections, and summarize relevant projects in Georgia and the Southeast. In addition, working groups conducted initial calculations of the solutions’ carbon-reduction potential within the state. This included an estimation of how the deployment of each solution could reduce net carbon emissions in the state by 1 Mt a year by 2030. This threshold of 1-Mt CO_2_-e represents almost 1% of Georgia’s 138-Mt CO_2_-e annual net GHG emissions, based on our baseline analysis.

The 75 solutions were then passed through a four-step downselect process.

#### Step 1: Is the Solution Technology and Market Ready for Georgia?

The first step of the downselection process was to drop solutions that were either (1) relevant on a global scale but not pertinent to Georgia or (2) not technology and market ready and, therefore, very unlikely to be deployed in Georgia by 2030. The 2030 timeline provides important guardrails for the types of solutions highlighted through this process. To deliver meaningful emission reductions in Georgia by 2030, solutions must be mature and cost-competitive technologies that are ready to be deployed in the state. This analysis does not attempt to predict all of the solutions that will be viable in decades to come due to declining technology costs and innovation.

For example, the solutions “improved rice cultivation” and “tropical rain forests” were dropped at this phase because Georgia is not a rice-producing state and does not have tropical rain forests. In addition, “autonomous vehicles” and “enhanced mineral weathering” to capture and store carbon in calcium and magnesium carbonates were dropped at this phase because they are unlikely to be ready for wide-scale deployment in Georgia by 2030.

#### Step 2: Is There Sufficient Local Experience and Available Data?

The remaining solutions were then evaluated on whether there is a track record of deploying the solution in Georgia or the southeastern United States. This metric provided an additional screen for local relevance as well as a proxy for predicting whether a project has the potential to be deployed in the near term. For example, the solution “offshore wind turbines” was dropped at this phase because no offshore wind farms have been built in Georgia or the Southeast to date. Similarly, “biochar” was set aside because the authors were aware of only one small-scale and recently established producer of it in the state of Georgia.

#### Step 3: Does the Solution Provide ~1-Mt Co_2_-e Reduction Annually by 2030?

Solutions must be able to provide meaningful emission reductions in Georgia by 2030. Solutions failed to meet this standard for a variety of reasons, including if the necessary infrastructure could not be built and operated in the 2030 timeframe, if they required substantial changes in near-term consumer preferences, or if they were poor matches for Georgia’s natural resources or economy. For example, the solutions “living buildings”, “net-zero buildings”, and “building with wood” were all dropped because the new construction of these buildings in Georgia in the next decade is not expected to be sufficient to meet the 1-Mt threshold. “Family planning” and “educating girls” also did not meet our threshold for the level of impact—these are generally considered highly impactful in transitional countries, but their impact is more limited in industrialized economies. “Nuclear” was dropped at this step because it is unlikely that additional new nuclear reactors could be permitted, built, and operated in Georgia by 2030. The analysis, however, does assume that the two nuclear units currently under construction at Plant Vogtle will be completed in the current decade.

#### Step 4: Is the Solution Cost-Competitive?

Finally, solutions must be cost-competitive with other solutions impacting the relevant sector. Each sector defined cost-competitiveness using customized metrics. For example, electricity sector projects were compared on their levelized cost of electricity, while forestry and land-use solutions were evaluated on the cost per metric ton (“tonne” or “t”) of sequestered CO_2_.

The result of this four-step downselection process is a set of 20 high-impact 2030 solutions for Georgia. These solutions could all contribute meaningfully to GHG emission reductions in the state. How these solutions are deployed, however, can impact societal priorities beyond carbon. As a result, a working group focused on how deployment could impact equity, public health, environment, and economic development. In this phase of analysis, the Beyond Carbon working group conducted an initial mapping of potential beyond-carbon impacts for each of the high-impact 2030 solutions, based on a literature review and a survey of experts.

## Results

**Fig. 5:**
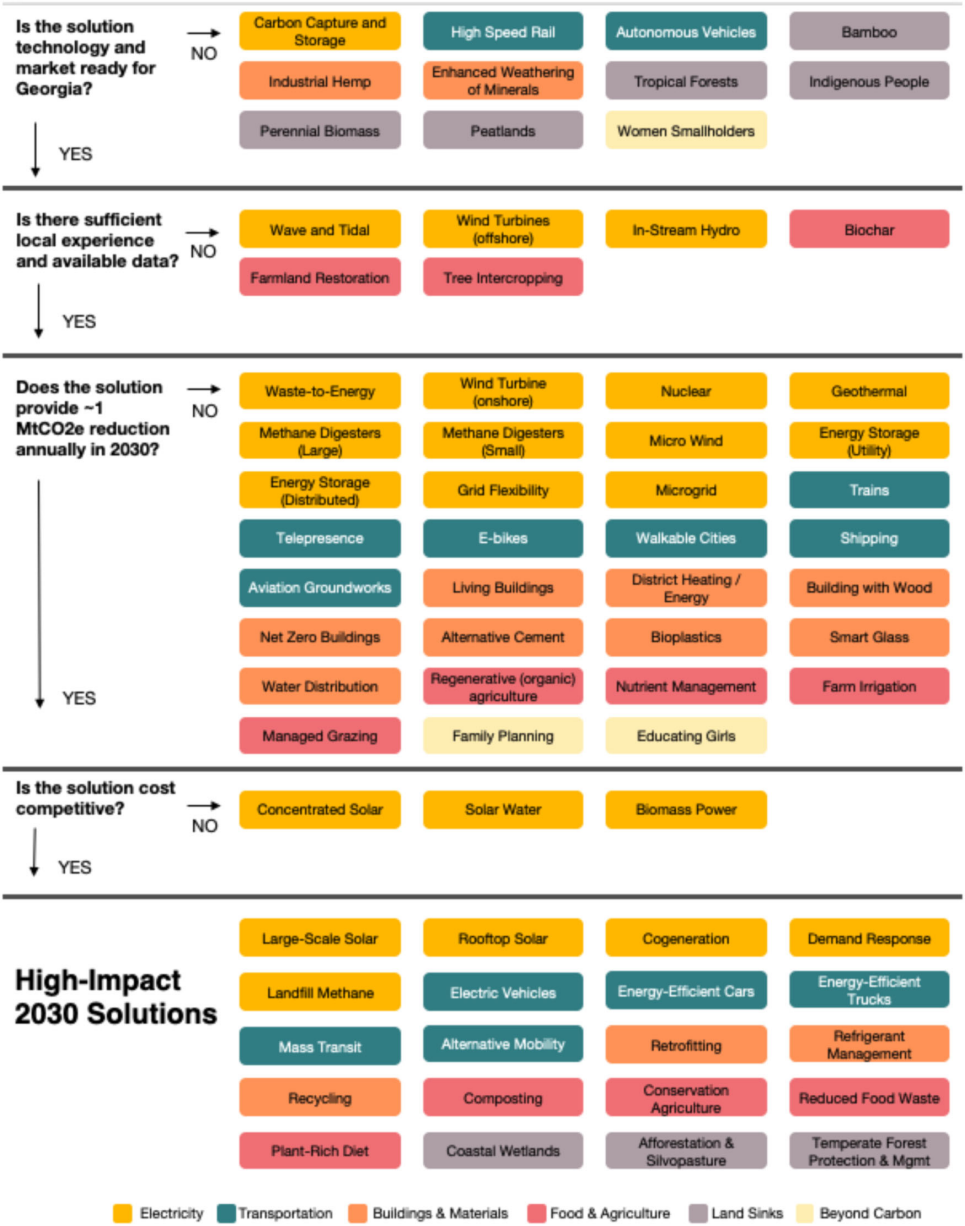
Drawdown Georgia downselect flowchart of solutions. This figure shows 71 solutions considered by Drawdown Georgia. We also considered “temperate forests”, “afforestation”, “forest protection”, and “silvopasture” as stand-alone solutions. These solutions passed the four-step downselect process; however, they were ultimately bundled with similar solutions and show up as part of the high-impact 2030 solution set. As result, they do not appear in the figure above. In total, Drawdown Georgia evaluated 75 solutions

The downselect results are summarized in Fig. 5. The figure shows at which point in the downselection process solutions dropped out of consideration, leading to the 20 high-impact 2030 solutions.

### Electricity Generation

Reflecting the forecast that 41% of Georgia’s energy-related CO_2_ emissions in 2030 will come from the electricity generation, this sector has five of the 20 high-impact 2030 solutions:

1. Cogeneration: cogeneration involves the coproduction of beneficial heat and electricity. It can involve capturing waste heat that is a by-product of coal- and gas-fired power production, where the captured heat can be used to heat water or buildings, manufacture products, or create more electricity. It can also involve the capture of waste heat from an industrial or commercial process that is then used to generate electricity, as done in the pulp and paper industry. Cogeneration reduces emissions by using waste heat to displace the consumption of fossil fuels that would have otherwise generated more emissions.
2. Demand response: demand response programs serve to “adjust the timing and amount of electricity use” and can help utility companies reduce peak load, shift load, or reduce overall usage. It changes electricity usage by end-use customers and encourages them to be responsive to changes in the price of electricity over time. It could take the form of incentive payments designed to induce lower electricity use when wholesale market prices are high or when system reliability is jeopardized.
3. Rooftop solar: solar photovoltaic systems convert solar energy into electricity. Rooftop solar systems are small-scale installations that can produce electricity primarily for on-site use. When combined with storage, additional benefits can accrue.
4. Large-scale farms: solar photovoltaic systems can convert solar energy into electricity. This solution includes solar farms, defined as any ground-mounted solar panel facility that has a capacity rating larger than 5 MW, as well as community-scale solar, which generally has a capacity of 0.5–5 MW. This solution also considers the possible advantage of coupled on-site storage to enhance reliability.
5. Landfill methane: landfills are a major source of methane emissions. Methane, a potent GHG, is created from anaerobic digestion of municipal solid waste in landfills. The gas can be captured and then used to generate electricity, which can prevent methane emissions and replace conventional electricity-generating technologies such as coal and natural gas.

**Fig. 6:**
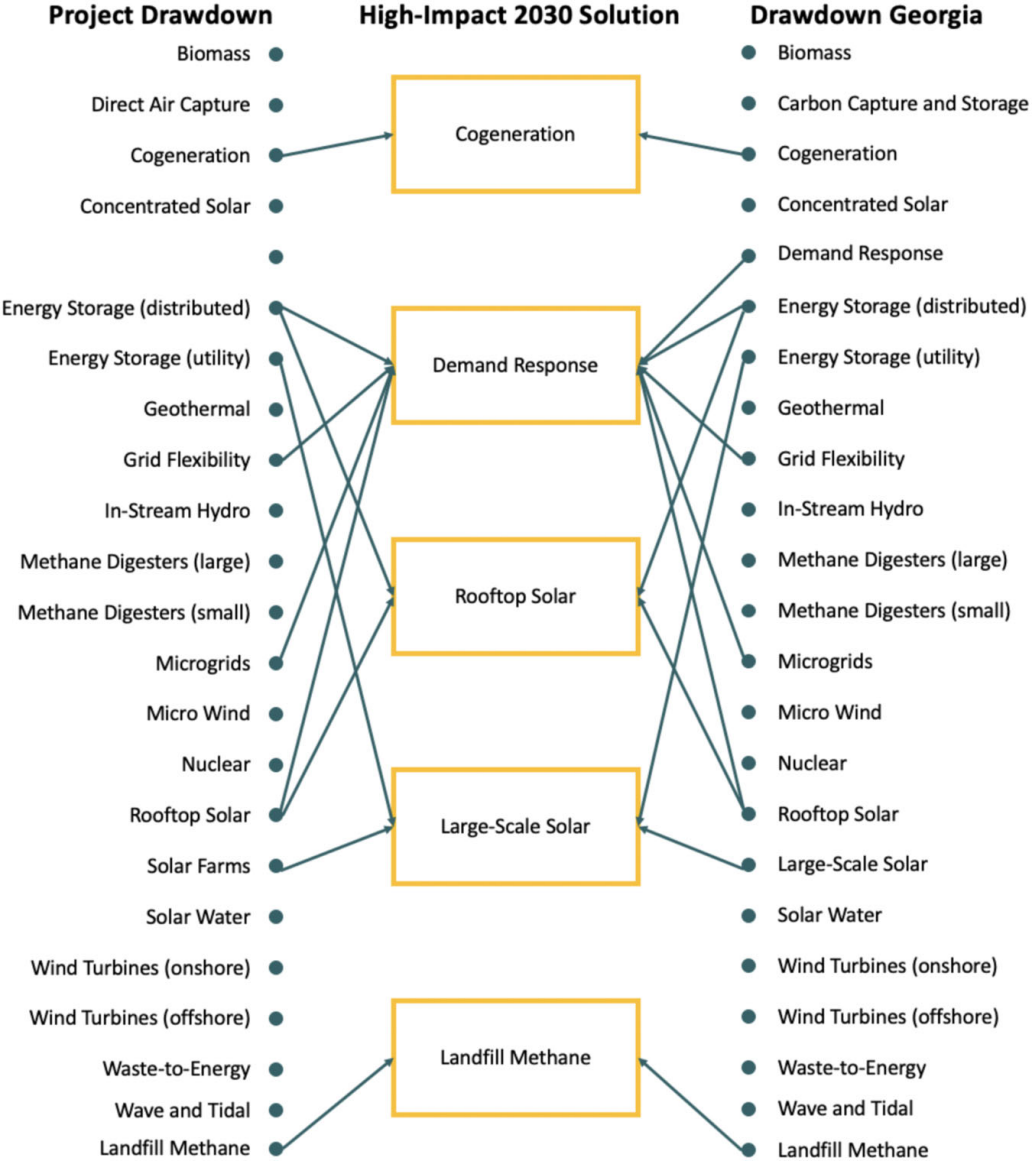
Crosswalk of drawdown solutions in the electricity sector. Artificial leaf, hydrogen–boron fusion, smart highways, and solid-state wave energy were “Coming Attractions” in Project Drawdown that were judged “out of scope” for Drawdown Georgia

These four solutions were derived from evaluating the 22 electricity sector solutions shown in Fig. 6. Note the addition of one solution—demand response—that was not included in the Project Drawdown list. Demand response is a bundled solution with features drawn from four of the original electricity solutions (see the arrows from the left). Cogeneration is the only high-impact 2030 solution that is not bundled with other electricity solutions. In addition, one of the Project Drawdown solutions, “direct air capture”, was renamed “carbon capture and sequestration” to consider a broader range of technologies available for capturing CO_2_ such as bioenergy carbon capture and storage.

Each of these 22 solutions was filtered through the four-step downselection process.

The requirement that each solution is able to provide at least 1-Mt reduction annually by 2030 caused 11 of the 22 solutions to drop out of consideration. This was based on preliminary estimates of carbon-reduction potential. These estimates showed an interesting span of new investments that would achieve 1 Mt of reductions from the electricity sector in Georgia. The five high-impact solutions would generate a megaton as follows:

1. Ten solar farms (@100 MW) and 36 community solar projects (@5 MW).
2. Sixteen factories capturing waste energy to cogenerate at least 25 MW of electricity.
3. In all, 295,000 home solar systems @5 KW.
4. Four typical landfill facilities with 5-MW gas-to-energy systems.
5. Overall, 187,000 households shift 10% of their peak electricity use to off-peak.

The other solutions would generate a megaton as follows:

1. Four parabolic trough-concentrated solar power plants.
2. Ten biomass power plants, each @50 MW, burning biomass waste.
3. Two hundred and sixteen typically sized methane digester projects.
4. One thousand two hundred and twenty-seven local geothermal energy projects.
5. In all, 215,000 microwind turbines.
6. Overall, 294,000 in-stream hydrogenerators.
7. In total, 7.1 million homes (70% of Georgia’s households) with solar water heating.

To illustrate the downselect methodology in more detail, consider solar farms and community solar. Georgia had <1 GW of utility-scale solar in 2017, and it is forecast to have <2 GW in 2020, growing to 4 GW in 2030. An additional 1 Mt of emissions could be avoided if an additional 1180 MW of utility-scale solar were to be constructed in Georgia, and operated at a 25% capacity factor in 2030. These additional solar facilities would occupy ~15 square miles of land, which is <0.03% of Georgia’s land. At the same time, local jobs would be created, and there would be rents to landowners, taxes to local municipalities, less air pollution, and public health benefits.

### Transportation

Reflecting the forecast that 39% of Georgia’s energy-related CO_2_ emissions in 2030 will come from the transportation sector, the list of high-impact 2030 solutions includes five transportation solutions:

1. Electric vehicles: electric vehicles are powered by electric batteries instead of conventional fuels such as gasoline and diesel. The emission profile of these vehicles is lower in both CO_2_ and other pollutants. That said, the exact emissions vary depending on the generation mix providing the electricity. The average CO_2_ intensity of electric power in Georgia has been on a downward trajectory due to coal retirement. This declining trend is expected to continue through 2030 owing to additional fuel switching, as well as increasing shares of generation from solar PV and nuclear power.
2. Energy-efficient cars: a range of cost-effective technologies are available to reduce or replace petroleum fuel use in light-duty vehicles (LDV), including cars and pickups. Among these, hybrid cars deliver the most substantial reductions by pairing an electric motor and battery with an internal combustion engine. The combination enables the vehicle to regenerate braking loss and operate both the engine and motor at greater efficiency, improving fuel economy and lowering emissions. Other technologies, such as lightweighting, advanced transmissions, and downsizing with turbocharging, promise to further reduce the CO_2_ intensity of LDV in Georgia.
3. Energy-efficient trucks: US trucks consume about 50 billion gallons of diesel fuel each year. Trucks consume a disproportionate quantity of fuel relative to distances traveled. Increasing fuel efficiency for both new and existing trucks can lead to significant emission reductions. Numerous fuel-saving technologies are available at compelling paybacks.
4. Public/mass transit: public mass transit includes modes such as buses, trains, and streetcars. When people rely on mass transit instead of cars, it reduces GHG emissions.
5. Alternative mobility: replacing emission-intensive VMTs with zero- or low-carbon alternatives, such as bicycling, walking, or teleworking, can reduce GHG emissions.

**Fig. 7:**
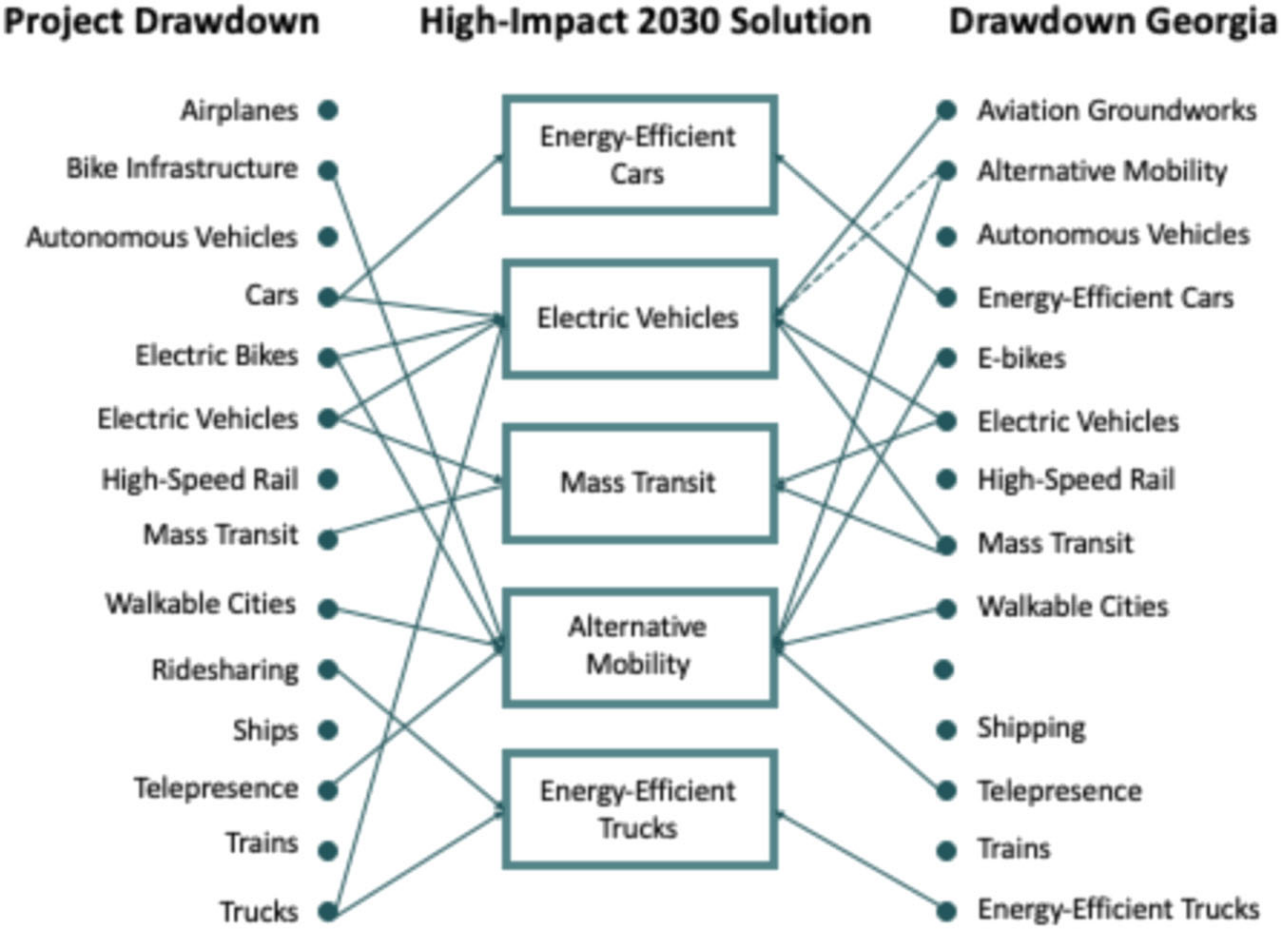
Crosswalk of Drawdown solutions in the transportation sector. Solid lines represent direct linkages and dashed lines represent indirect linkages

These solutions were part of the 13 transportation sector solutions evaluated by Drawdown Georgia (Fig. 7).

The “alternate mobility” solution is a bundle of related solutions that Drawdown Georgia is considering as a group, including bike infrastructure, walkable cities, telepresence, e-bikes, and e-scooters, with a specific focus on replacing short-distance vehicle trips with these alternatives. Our analysis of this solution was completed before the coronavirus pandemic, which greatly expanded telepresence adoption in Georgia and across the country. Future analysis will consider the emission impact of expanded telepresence, as well as how telepresence adoption rates in Georgia might change post pandemic.

Each of these transportation technologies was filtered through the four-step downselection process. Two solutions were dropped out for lack of market readiness (autonomous vehicles and high-speed rail) and five for not meeting the 1-Mt reduction minimum (e-bikes, shipping, telepresence, trains, and walkable cities). E-bikes and telepresence in the end were bundled with walking and biking into “alternative mobility” in the built environment and materials sector.

To illustrate the downselect methodology, consider electric vehicles, which are gaining market share nationwide and in Georgia. The higher price of EVs remains a deterrent, but prices are dropping, with some estimates suggesting parity on a total cost of ownership basis within a decade. Further cost reductions and policy stimulation may be needed to accelerate the transition so that 1 Mt can be diverted annually by 2030. The decarbonization of the grid is improving the environmental benefits of EVs, and managed charging (in which EV charging needs are considered in view of the power sector’s hourly dispatch) can help optimize the economic benefits of EVs to the utility. In general, the addition of more nuclear power will facilitate comparatively lower CO_2_ charging during off-peak hours, and solar additions can contribute to lower CO_2_ recharging during early afternoon hours. 1 Mt of avoided emissions could be achieved in Georgia if 250,000 gasoline-powered vehicles were replaced with EVs in 2030, representing 2.9% of the state’s total fleet of LDV and accounting for about 10.7% of new LDV sales in 2030. At the same time, there would be improved air quality and fuel expenditure savings, as well as a total cost of ownership for EVs that is approaching cost parity. This scenario is compared to a baseline that assumes business as usual for fuel economy and CO_2_ reductions driven by new vehicle technologies and Federal CAFÉ regulations, as well as an electric grid that will continue to decarbonize with additional reliance on natural gas and renewables.

### Built Environment and Materials

**Fig. 8:**
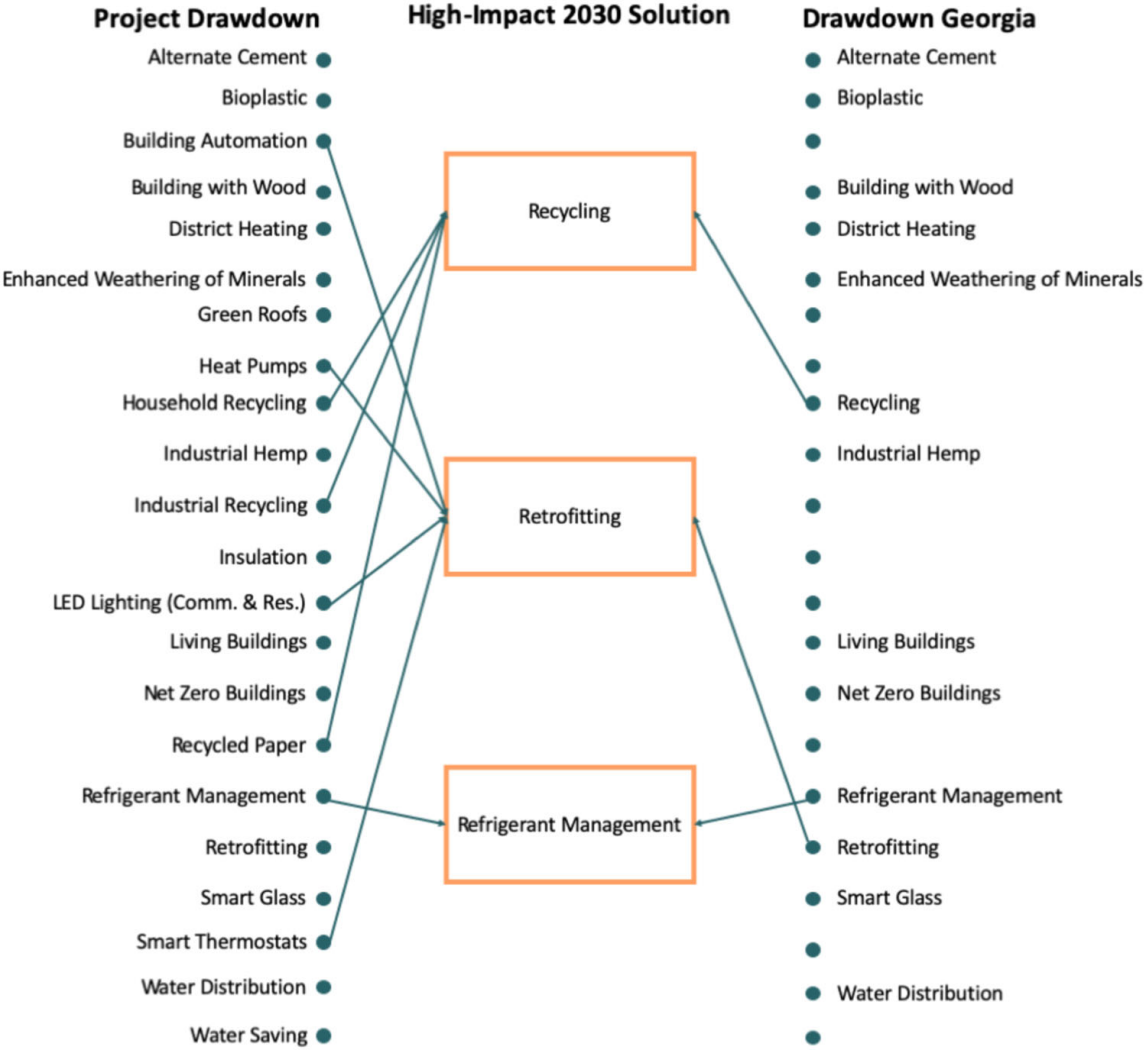
Crosswalk of Drawdown solutions in the built environment and materials sector

The list of high-impact 2030 solutions includes three solutions from the built environment and materials sectors, reflecting the fact that 43% of carbon emissions from energy consumption in Georgia in 2030 are expected to come from equipment used in residential and commercial buildings:

1. Recycling/waste management: recycling can reduce GHG emissions because it is often less energy-intensive than producing new items. This solution considers increases in recycling at the household level, industrial and commercial recycling, and paper recycling.
2. Refrigerant management: hydrofluorocarbons (HFCs) are chemicals used to cool refrigerators and air conditioners. They are also an extremely potent GHG. Efforts to control leakages and replace HFCs with alternative refrigerants and to properly dispose of and recycle existing HFCs would lower GHG emissions.
3. Retrofitting: buildings use electricity and natural gas for heating, ventilation, and cooling (HVAC), water heating, lighting, and to power appliances and electronic devices. Retrofitting existing buildings to reduce energy demand can lower the GHG emissions due to these energy uses (Fig. 8).

The retrofitting bundle is the most complex of the bundled solutions. It includes three solutions from Project Drawdown, and a range of additional retrofit components, including:

1. Improving the insulation of existing buildings.
2. Replacing conventional lighting with LED lighting in both residential and commercial buildings.
3. Replacing conventional HVAC systems and gas- and oil-fired furnaces with high-efficiency heat pumps.
4. Installing water-saving devices such as low-flow fixtures and efficient appliances.
5. Replacing conventional thermostats with smart thermostats.
6. Using automated control systems in existing commercial buildings that can regulate heating, cooling, lighting, and appliances to maximize energy efficiency.
7. Using alternative roof designs such as green roofs, which line a roof with soil and vegetation, as well as cool roofs, which reflect solar energy to reduce a building’s electricity demand and therefore reduce emissions.

To model this bundle of solutions, a focus group of experts was assembled and consulted on the cost-effective approaches to retrofitting without significant government intervention, acknowledging that some government intervention might be needed, but remaining agnostic to the specific interventions that might be most effective. Different subsets of the technologies were modeled based on expert opinion and a modeling approach that constrained investments in the space to cost-effective solutions. We defined cost-effective solutions as individual solutions that had a positive NPV at a 12% discount rate for our low estimate, and a bundle of solutions that had a collective positive NPV at an 8% discount rate. We estimated potential penetration rates based on performance of state-level retrofitting programs.^4^

Project Drawdown’s strong ranking of carbon drawdown potential for refrigerant management is notable because this mitigation solution receives relatively limited attention in the popular press and academic literature. Its high global warming potential highlights the need to manage non-CO_2_ sources of GHGs. This solution is also hampered by a lack of regulations and incentives for investments in improved performance, as well as limited information about management approaches, potential costs, and key stakeholders. In addition, many of the solutions considered in the built environment sector have diffuse impacts on GHG emissions. Without steps to consider joint technologies or to bundle technologies, few energy-efficiency technologies could individually make a sufficient impact on GHG emissions in Georgia by 2030. Finally, many of these solutions lack basic national or local data on the extent of their practices and associated costs. This lack of information makes defining an achievable scenario quite challenging.

To illustrate the downselect methodology, consider building retrofits. In 2017, Georgia’s commercial and residential buildings were responsible for emissions of 44.1-Mt CO_2_-e, and the baseline forecast suggests only a small reduction to 43-Mt CO_2_-e in 2030. An additional megaton of emissions could be avoided in 2030 by retrofitting around 20% of Georgia’s single-family residential homes (~600,000 homes) to achieve average energy savings of 20% per home by 2030. At the same time, energy bills and burdens would be lower, local jobs would be created, and there would be less air pollution.

### Food Systems

The list of high-impact 2030 solutions includes four solutions from the food systems sectors:

1. Composting: when organic matter decomposes in landfills, it releases methane, a potent GHG. Composting allows for organic matter to be broken down by microbes. The process sequesters carbon and produces fertilizer.
2. Conservation agriculture: conservation agriculture refers to a bundle of agricultural practices that supports biosequestration via crop rotation, cover cropping, and reduced tillage.
3. Plant-rich diet: a plant-rich diet, such as a vegetarian or vegan diet, would reduce emissions associated with meat production. This solution assumes that people (1) maintain a 2500-calorie-per-day nutritional regime, (2) meet daily protein requirements, and (3) purchase locally produced food when available.
4. Reduced food waste: food waste refers to food that is produced but not eaten.

**Fig. 9:**
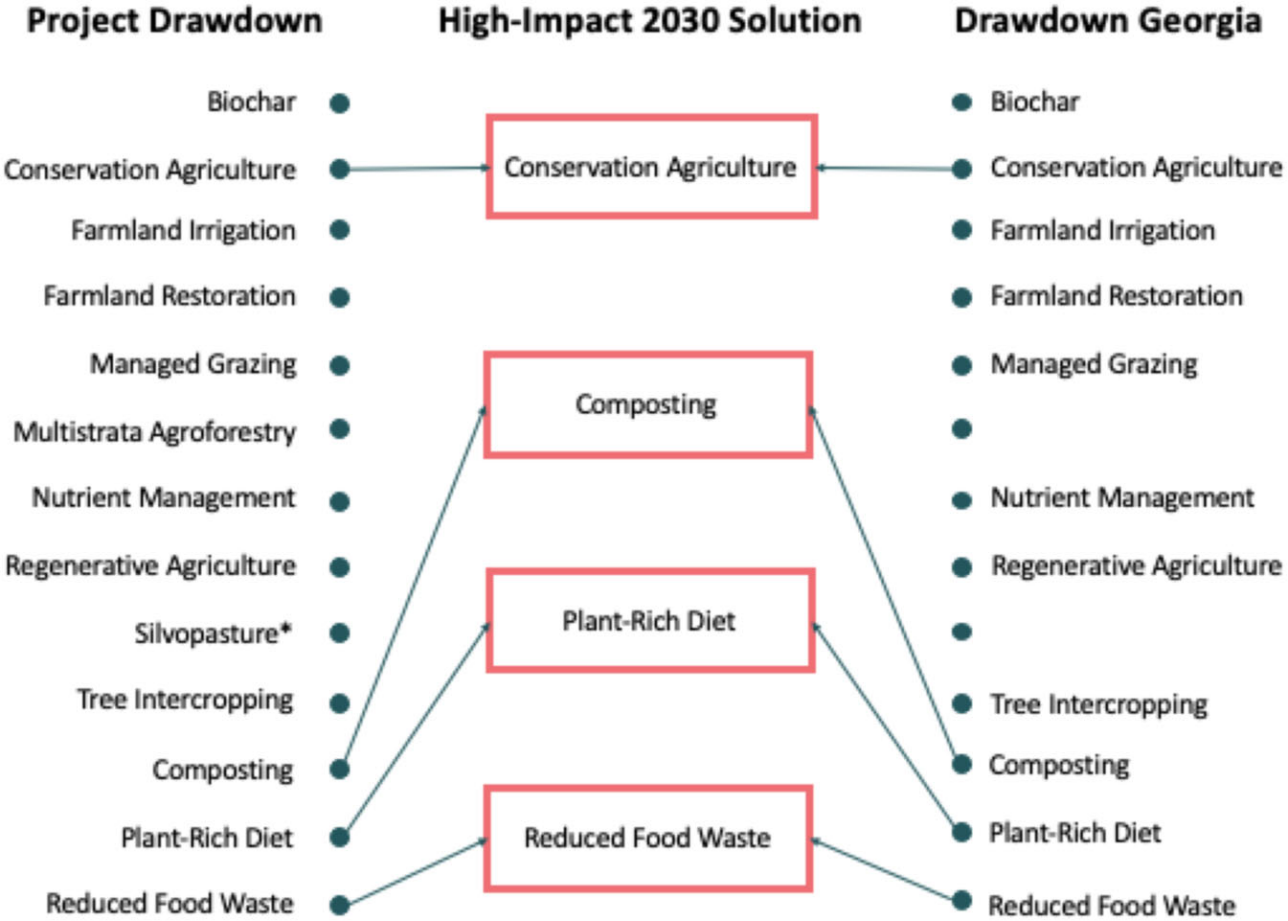
Crosswalk of Drawdown solutions in the food systems sector. *See Land Sinks

These solutions were derived from the 11 food system solutions evaluated by Drawdown Georgia (Fig. 9). Conservation agriculture comprises five related Project Drawdown solutions that we treat as a bundled group.

To illustrate the downselect methodology, consider reduced food waste, which is an emission-reduction opportunity that has gained local as well as national attention. The United States produces about 61 Mt of annual food waste along the entire food supply chain. Waste can occur for a variety of reasons, such as people purchasing more food than they need or customers rejecting bruised or misshapen produce. Food losses also can occur when food rots on farms or is not harvested to meet market demands.

While food losses and waste generate GHGs in every step of the food production, manufacturing, and distribution process, more than 50% of the food waste occurs at the retail and consumer levels (Buzby and Hyman 2012; ReFED 2016). When the food waste ends up in the landfills, they release methane, a potent GHG. Based on the per capita food waste generation in the United States, Georgia generates about 2 Mt of food waste, which is equivalent to the total GHG emissions of about 8-Mt CO_2_-e in 2017. If Georgia could prevent about 12% of the current food waste (equivalent to about 0.25 Mt/year), about 1 Mt of emissions can be achieved in 2030. At the same time, local jobs would be created, food chains would receive tax benefits, food insecurity would be improved, and there would be less air pollution.

### Land Sinks

The list of high-impact 2030 solutions includes three solutions from the land sinks sector:

1. Afforestation and silvopasture: afforestation is the process of promoting forests in places that currently have no forests yet were historically forested and capable of sustaining forests. This could include planting trees on degraded agricultural or on pasture lands (i.e., silvopasture) and planting in urban areas. Forests sequester carbon in trees, soil, and other vegetation.
2. Coastal wetlands: coastal wetlands, including seagrasses, tidal salt marshes, and freshwater marshes, are effective carbon sinks. These ecosystems sequester carbon in plants and particularly in sediments.
3. Temperate forest protection and management: restoring and managing temperate-climate forests has many benefits, including carbon sequestration from trees, soil, and other vegetation. Protecting existing forests, including old-growth forests, can reduce deforestation rates and safeguard carbon sinks. This includes legal protections as well as market-driven programs.

**Fig. 10:**
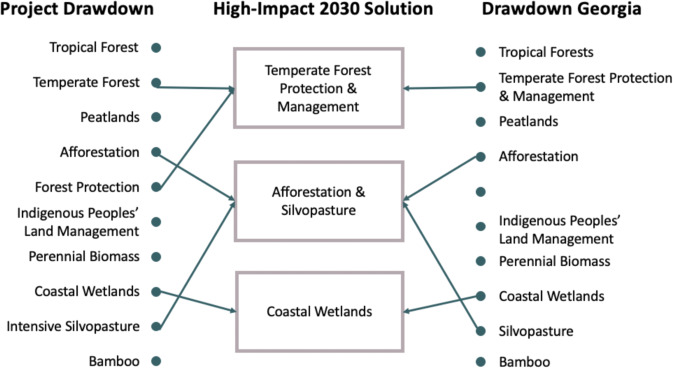
Crosswalk of Drawdown solutions in Land Sinks

These solutions were derived from the 11 forestry and land-use solutions evaluated by Drawdown Georgia (Fig. 10).

Georgia’s forests are already a large-scale carbon sequestration ecosystem. Based on Forest Inventory and Analysis data, between 2007 and 2017 Georgia forests accumulated an average of 27 Mt annually in living tree biomass above and below ground.^5^ A preliminary estimate of annual carbon uptake in state soils is 1–3 Mt (Richter et al. 1999; Carey et al. 2016; Crowther et al. 2016; Machmuller et al. 2018). This brings the total estimated annual carbon sequestration of Georgia’s forests to an estimated 30-Mt CO_2_.

More carbon sequestration in the forests and soils of Georgia is possible. To illustrate, consider the use of afforestation and silvopasture to increase the amount of CO_2_ that is sequestered in trees and soils in Georgia. Currently, very little crop and pasture land in Georgia is planted below trees. If 7% of the state’s current pasture acreage were planted with mixed tree species (loblolly pine and mixed hardwoods), an additional megaton of CO_2_ storage could be achieved by 2030. At the same time, the health and productivity of livestock would be improved, biodiversity would expand, and there would be improved stream water quality.

### Women and Girls

Project Drawdown highlighted three solutions in this category: women smallholders, family planning, and educating girls. None of these solutions were retained by Drawdown Georgia. Women smallholders were deemed out of scope. The latter two solutions were dropped for two reasons. First, the high global impact of these solutions noted by Project Drawdown rests on developing countries where large-scale gaps in these areas and high fertility rates offer material opportunities for achieving carbon-reduction objectives. In contrast to developing countries, population growth in the United States is relatively low and appears to be slowing overall (Livingston 2019). Consequently, these solutions do not reach the emission-reduction threshold of 1-Mt CO_2_-e. Second, there is an important beyond-carbon dimension to consider. Black and brown communities account for over 90% of US population growth (Passel et al. 2012). Therefore, viewing choices about the number of children a family has through the “carbon” lens can create a disproportionate and negative focus on families of color and reinforce a dynamic that problematizes reproductive decisions by women in general, and by women of color in particular. This is an important equity issue and further reinforces the decision to eliminate these solutions from the list of high-impact solutions for Drawdown Georgia.

Nevertheless, our work recognizes the important role for women and girls in reducing Georgia’s carbon emissions. Addressing gender and racial gaps in US education (e.g., engagement in STEM fields) may offer some opportunities for carbon reduction (Cordero et al. 2020). Moreover, research suggests that women in decision-making roles at organizations tend to make more sustainable choices than their male counterparts (Ben-Amar et al. 2017), which speaks to a major role for leadership opportunities for women, both in general and in Drawdown Georgia implementation.

### Assessment of Beyond-Carbon Attributes

Public engagement and qualitative, multicriteria assessments of “beyond-carbon” attributes were conducted to provide insights on how to implement solutions that are sensitive and flexible to local equity, environmental, health, and economic contexts. Public engagement was weaved throughout the “beyond-carbon” process (i.e., surveys, stakeholder meetings, discussion sessions, and public forums) with nonprofit and community stakeholders representing diverse social and environmental issues. Implementation of the 20 high-impact 2030 solutions will entail a range of impacts and benefits beyond-carbon mitigation. We organized these impacts and benefits into four “beyond-carbon” categories: environment, equity, economic development/jobs, and public health. Within each category, we further identified the main dimensions to consider (Table 1).

**Table 1:** Beyond-carbon attributes and dimensions

Environment	Equity	Economic development/jobs	Public health
• Air quality	• Affordability	• Local economy and employment	• Premature mortality
• Water quality	• Workforce/business diversity	• Input prices/system costs	• Morbidity
• Land use	• Distribution of public health impacts	• Workforce job quality	• Quality of life
• Ecosystem/biodiversity	• Accessibility	• Wages and benefits	• Education
• Material disposability	• Cultural fit and way of life	• Property values and taxes	• Public safety
		• Infrastructure requirements	

**Fig. 11:**
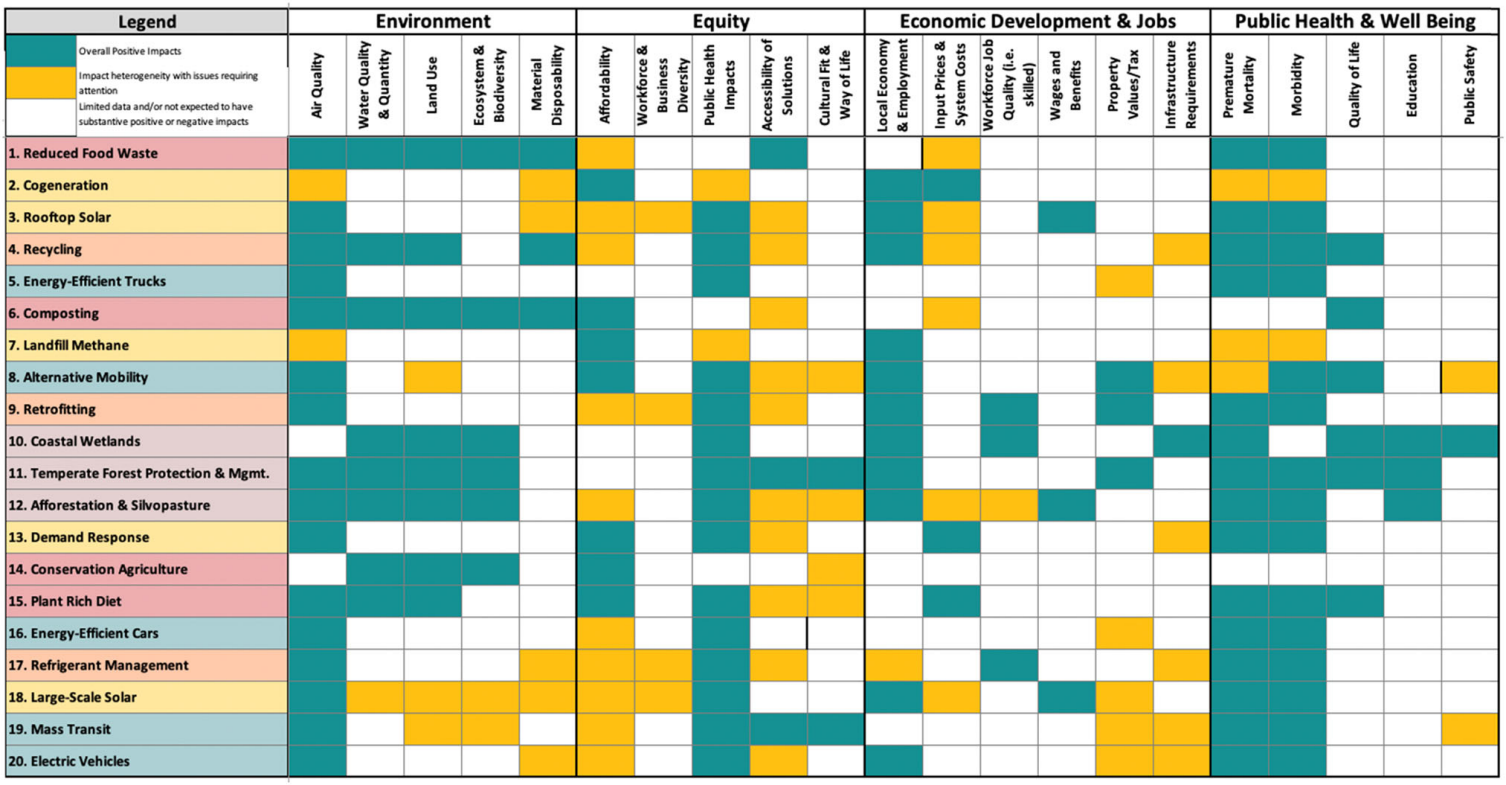
Outcome of qualitative multicriteria assessment for selected attributes

A number of key observations can be made for each category based on a qualitative, multicriteria assessment of the high-impact 2030 solutions (the assessment outcomes for a subset of attributes are presented in Fig. 11). A more detailed qualitative and quantitative beyond-carbon analysis will be included as part of the next phase of work.

### Environment

Significant air-quality improvements are one of the primary benefits associated with the vast majority of solutions and these result in a range of public health benefits (noted below). In addition, food systems as well as land sink solutions, offer positive water quality and usage impacts as a result of solution efficiencies and reduced soil erosion. Conservation agriculture practices and temperate forest stewardship extend the benefits of our natural carbon sinks like trees, soil, and other vegetation. In turn, a healthier natural world hedges against extreme weather impacts. In addition to benefits, there are also some impacts to be managed, including the disposition of hazardous materials (e.g., batteries and panels) in the solar, cogeneration, and electric vehicle solutions and land/water use and related biodiversity issues associated with large solar farms.

### Equity

We consider equity issues through several lenses. Land use and environmental justice issues associated with current US power technologies are comprehensively covered by Massetti et al. (2017), including climate vulnerability impacts on underresourced communities. A transition to the electricity generation solutions in Drawdown Georgia promises improvements in many of these areas. At the same time, the equity lens also considers the extent to which different communities, particularly underresourced populations, will have access to, or benefit directly from, solutions. While many communities will derive benefits via air-quality improvements and public health outcomes, some solutions such as cogeneration have the potential to adversely impact local air quality, depending on plant controls and location (Yang et al. 2019). In addition, affordability of some solutions such as solar panels or silvopasture farming presents barriers to solution access (and enjoyment of the corresponding advantages). Other solutions have the potential, if not implemented with equity in mind, to perpetuate current equity challenges that go deeper than affordability. For example, Sunter et al. (2019) found racial and ethnic differences in rooftop solar adoption in the United States, even after accounting for income and household ownership.

Similarly, rate design to recover fixed utility costs due to lower electricity consumption after residential PV penetration and large-scale retrofitting can potentially exacerbate the “energy burden” experienced by lower-income households who purchase all of their electricity from the grid (Bird et al. 2015; Johnson et al. 2017). Finally, some of the businesses and workforce supporting these solutions currently lack diversity—thereby presenting an opportunity to extend benefits via direct and indirect jobs and wealth-building to a wider swath of underrepresented individuals and groups.^6^

### Economic Development and Jobs

The vast majority of the solutions present job opportunities—for forest managers, waste management personnel, solar installers (The Solar Foundation 2019), construction workers, and home retrofit contractors (Brown et al. 2020). However, there can be displacement impacts, as these solutions replace current practices and technologies that would be phased out. In addition, while reducing food waste and composting are likely to have positive economic benefits overall through increased efficiencies, there can be increased input or system costs for farmers or solution owners and corresponding price pressures in the value chain. Similarly, infrastructure costs for electric vehicles and mass transit will need to be addressed. On the other hand, given the increased cost-competitiveness of many of the solutions, such as solar farms and community solar, compared to existing alternatives, we do not envision significant adverse impacts on energy prices (though note above comments on energy-burdened customers). For other solutions, there may be property value impacts (positive or negative) depending on siting decisions.

### Public Health

Many of the solutions offer direct and indirect material public health benefits on mortality and morbidity (e.g., asthma and mental impairment) rates. These benefits are largely related to projected improvement in air quality and are most pronounced in the electricity, transportation, built environment, and several of the forestry solutions. In addition to these impacts, many solutions, including those in the agricultural and food system areas, also offer diverse benefits that impact quality of life, education, and public safety. Some solutions contributing overall benefits may have local impacts such as cogeneration (local air quality) and mass transit (public safety) that will require attention.

## Discussion

While this initial phase of the Drawdown Georgia project has achieved a great deal, more work is needed. This concluding section begins by discussing the strengths and limitations of the downselect process used to identify high-impact 2030 solutions for Georgia. The paper ends with a short discussion of planned next steps.

### Validity

This working paper documents the first assessment of the Project Drawdown’s 102 global solutions in terms of their applicability and potential if implemented in an individual US State. By developing, executing, and documenting a rigorous and replicable methodology for identifying high-impact solutions for 2030, Drawdown Georgia paves the way for other states to jumpstart similar assessments.

As other states consider replicating this process, the strengths and weaknesses of Drawdown Georgia’s downselection process must be considered. Key among the strengths of Drawdown Georgia is its use of public domain data and publicly available analytical tools. The authors of this paper are all academics with no conflicts of interest that might cause bias in the design and conduct of this study.

Another strength of Drawdown Georgia is its innovative assessment of bundles of solutions that more closely align with decision-making institutions at the state and local levels. Without bundling, the use of a 1-Mt minimum threshold would have precluded many modestly impactful technologies that, if implemented today, could lead to significant reductions on their own by 2030. In an effort to not exclude numerous small-scale solutions, collections of solutions were considered. For example, retrofitting of existing buildings includes a group of solutions, such as improving building automation, insulation, recommissioning, and installing LED lighting. These solutions, while not as effective individually in contributing to the 1-Mt threshold, are able to make significant reductions when considered together.

A third strength is that, by highlighting actions that can deliver impact by 2030, we are offering policymakers and practitioners a menu of solutions that can be implemented in the very near term, which is increasingly important in light of the scientific community’s findings that we need to act quickly to achieve even the 2 °C target, let alone the 1.5 °C target.

On the other hand, there are at least four limitations that warrant consideration as our findings are examined by stakeholders in Georgia and elsewhere.

First, the downselection process emphasizes the ability of solutions to deliver carbon reductions by the year 2030. This timeframe excludes solutions that may not be technologically or market ready in Georgia in the near term, but have real potential to play a meaningful role in later decades. This includes solutions such as offshore wind and direct air capture of CO_2_. Our focus on the near term should not divert attention away from the need to consider long-term solutions going forward. Other solutions are too small to meet the 1-Mt threshold individually, and bundling is not a logical solution. Examples are the construction of zero-energy buildings and the use of engineered wood in construction: it is unlikely that enough new buildings will be constructed by 2030 to meet the emission-reduction threshold. Similarly, the widespread use of biochar in crop or marginal lands with an affordable price tag will unlikely store enough carbon in the soil by 2030. Managed and regenerative grazing of livestock could offer low-carbon, meat-based diet to people, but such a solution requires decades of commitment to regenerative farming practices.

We recognize that today’s challenges are largely a product of past investment patterns and caution that near-term solutions may “lock-in” and pose barriers to the deployment of superior longer-term, transformative changes (Markolf et al. 2018, Brown et al., 2008). The technologies introduced over the next decade will become incumbent technologies with newly created support system that will make future transitions more difficult. For instance, natural gas cogeneration replacing coal-fired electricity over the next 10 years would reduce GHG emissions, but it could also lock in future emissions from natural gas technologies that could otherwise have eventually progressed to net-zero technologies such as renewables. Thus, it is important to be attentive to emerging technology trends and consider ways to facilitate and accelerate future transitions.

Second, examining each Drawdown solution in isolation can lead to over- or underestimates of carbon-reduction potential. A systems approach is critical to understanding the net impacts of multiple carbon mitigation actions.

Some solutions are “synergistic”. Here, successful deployment of one solution can magnify the carbon-reduction potential of another solution. On the one hand, there could be “emissions synergies” in which implementation of one solution (e.g., large-scale solar) boosts the emission-reduction potential of another (e.g., electric vehicles powered by a lower-carbon electric grid). On the other hand, there could be “implementation synergies” in which implementation of one solution (e.g., afforestation and silvopasture) can speed up or ease the implementation of another solution (e.g., coastal wetlands, which are healthier because of the pollution filtering of upstream forests).

Solutions can also be “competitive”. Here too, there can be “emissions competition”, in which implementing one solution (e.g., large-scale solar) reduces the emission reductions that can be achieved by another (e.g., building retrofitting, because the electricity that would be “saved”, would not be as carbon intensive). There can also be “implementation competition”, for example, when the successful reduction of food waste and the adoption of composting reduces organic matter at landfills, thereby reducing opportunities for landfill methane projects. Thus, there is a temporal dynamic to the rise and decline of individual solutions. Solutions can also compete for limited acreage in Georgia—e.g., for planting trees or building solar farms. As a result, strategic deployment of these solutions will be critical. Innovative siting options will be needed, such as The Ray’s pilot solar array on highway rights-of-way along West Georgia’s I–85 (https://theray.org/). Innovative approaches to conflict resolution and citizen engagement may also be particularly valuable going forward. Future research needs to examine key social–ecological–technological system interactions (Markolf et al. 2018, Brown et al., 2008). Optimizing solution impacts to include beyond-carbon benefits can enable transitioning to a more sustainable economy and healthier future generations.

Third, our analysis to date does not consider all of the potential leakage or life-cycle impacts of each Drawdown solution that can occur outside of Georgia. Perhaps, the simplest example of possible carbon leakage is if a Drawdown solution were to increase energy prices in Georgia. If this change results in an energy-intensive industry relocating to another state with a more carbon-intensive energy system, then the net savings of the solution should be diminished, but we do not make such an adjustment. A first step toward addressing this limitation would be to consider whether the emissions occur in Georgia or out of state (i.e., deemed emissions or logistic emissions), as well as whether they are the result of goods and services consumed in the state (i.e., direct emissions) or out of state (i.e., responsible emissions) (Sovacool and Brown 2010). In national accounting of carbon metrics, the IPCC distinguishes between territorial-based and consumption-based approaches (IPCC 2014, Fig. 5.14). The approach used in the Drawdown Georgia assessment is more territorial than consumption-based, although inconsistencies occur because necessary data and modeling tools are sometimes unavailable.

Finally, the Project Drawdown approach is fundamentally focused on the potential for cost-competitive reductions of net carbon emissions. In Drawdown Georgia, we expanded this framework by systematically identifying material “beyond-carbon” considerations. However, we recognize that the list of top 20 solutions may have been different if the primary solution selection criterion was not reducing carbon, but rather maximizing health impacts, promoting environmental and social justice, or optimizing job creation potential. In addition, our “beyond-carbon” analysis is qualitative and does not provide a quantification of beyond-carbon costs and benefits. As such, it may have resulted in the selection of high-impact 2030 solutions that have significant co-costs as well as the elimination of solutions that have significant cobenefits. Subsequent analysis is needed to determine the magnitude of this limitation.

### Beyond-Carbon Considerations

Implementation of the 20 high-impact 2030 solutions will have impacts and benefits beyond-carbon mitigation. How these solutions are deployed can support or hinder other societal priorities such as the broader environment, equity, economic development, jobs, and public health. Procedural equity, the broad inclusion of stakeholders in policy decision-making and implementation (Foster et al. 2019; Brown et al. 2020), will be particularly important in addressing the needs of communities in Georgia who are most vulnerable to climate change. Ongoing analysis focused on these beyond-carbon considerations is intended to highlight potential impacts and flag examples of best practices.

In the wake of the coronavirus pandemic, it is particularly important to focus on how the deployment of the high-impact solutions can help subnational entities recover. The pandemic has stimulated discussions of a “new normal” such as teleworking, and has raised the prospect for economic stimulus packages targeted towards accelerating a low-carbon transition. As discussed earlier, many of these solutions present job opportunities in different sectors; their job displacement impacts also need to be considered as part of any implementation assessment.

## Conclusions and Future Research

Although we have identified highly impactful near-term solutions, our preliminary analysis suggests that these solutions alone are highly unlikely to bring Georgia to carbon neutrality by 2030. Additional market penetration, technology advances, and new solutions will be needed in the 2040 timeframe and beyond to fully balance out Georgia’s sources and sinks of GHG emissions.

Our next phase of research involves a deeper analysis of the 20 high-impact 2030 solutions, including an exploration of the feedbacks and relationships among the 20 solutions, assessing the subsets that have strong synergistic or competitive effects. We will also be examining the benefits that go beyond carbon-emission reductions: providing new economic opportunities for residents of Georgia, advancing equity, supporting other environmental priorities, and improving public health. By understanding beyond-carbon attributes, Drawdown solutions also can be optimized in ways that will enable and stimulate their carbon-reduction impacts. Finally, our future research will identify barriers that hinder the adoption of the high-impact 2030 solutions as well as enablers and accelerators that might promote their utilization. This will lay the groundwork for implementation of supporting public–private partnerships, climate-friendly policies, citizen science and engagement, and a broad sweep of targeted initiatives. Continued engagement of experts and stakeholders, especially the business community, is vital in this next phase of work to encourage collective impact commitments.

Ultimately, we hope to inspire a transformational, evidence-based roadmap that will identify strategies for businesses, local communities, municipalities, and civic leaders across the state of Georgia—and across other subnational entities—to reduce their carbon footprints and strive for carbon neutrality.

## Figures and Tables

**Fig. 12:**
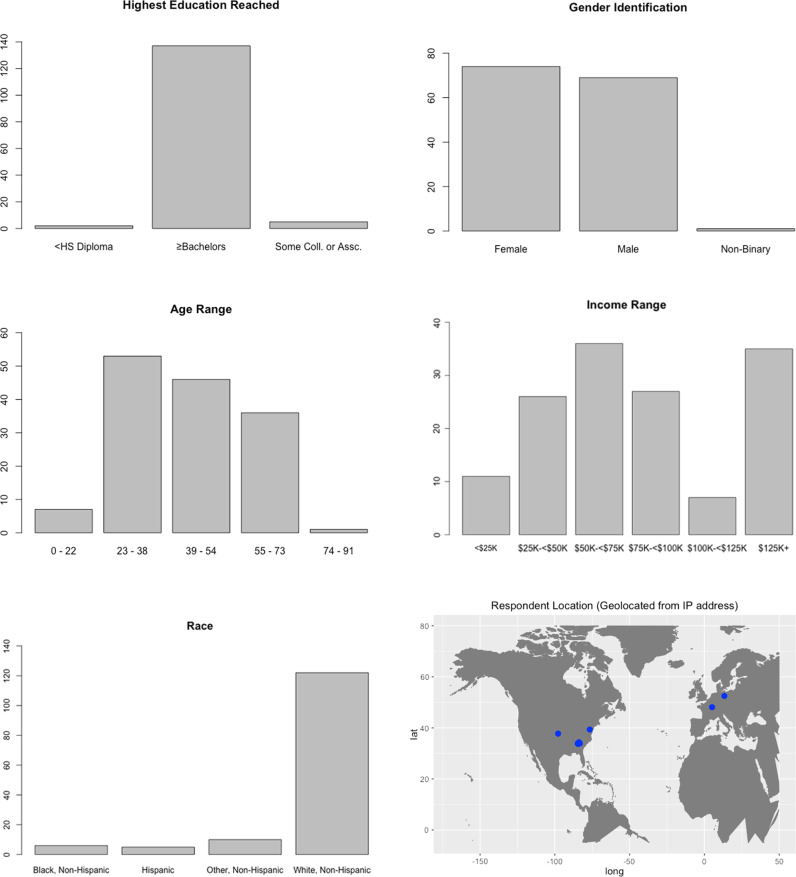
Demographics of the 280 respondents in the public survey

## Appendix

Fig. 12

## Footnotes

1 This reduces to ~130 Mt or 26% of the US total, if net exports from interstate flows are omitted.

2 The Beyond Carbon working group examined solutions from Project Drawdown impacting women and girls. They also examined Drawdown Georgia solutions for impacts “beyond carbon” including equity, economic development, public health, and the broader environment.

3 Additional solutions considered include high-efficiency windows, retrocommissioning of commercial buildings, and dead-band range expansion.

4 For further details, see the technical appendix posted here: https://cepl.gatech.edu/projects/Drawdown-Georgia.

5 Forest Inventory Analysis (FIA) USDA Forest Service. Knoxville, TN. Weblink: https://www.fia.fs.fed.us/.

6 For example, the 2019 Solar Jobs Census found that only 26% of the solar workforce was made up of women, and 73.2% of the overall solar workforce is white. Georgia ranks below national levels in terms of diversity, as women consist of only 18.9% of the solar workforce, and 76.6% of the workforce is white.
